# Running With Scissors: Evolutionary Conflicts Between Viral Proteases and the Host Immune System

**DOI:** 10.3389/fimmu.2021.769543

**Published:** 2021-11-01

**Authors:** Brian V. Tsu, Elizabeth J. Fay, Katelyn T. Nguyen, Miles R. Corley, Bindhu Hosuru, Viviana A. Dominguez, Matthew D. Daugherty

**Affiliations:** Division of Biological Sciences, University of California, San Diego, CA, United States

**Keywords:** viral proteases, host-virus evolution, innate antiviral immunity, molecular arms races, effector-triggered immunity, inflammasome

## Abstract

Many pathogens encode proteases that serve to antagonize the host immune system. In particular, viruses with a positive-sense single-stranded RNA genome [(+)ssRNA], including picornaviruses, flaviviruses, and coronaviruses, encode proteases that are not only required for processing viral polyproteins into functional units but also manipulate crucial host cellular processes through their proteolytic activity. Because these proteases must cleave numerous polyprotein sites as well as diverse host targets, evolution of these viral proteases is expected to be highly constrained. However, despite this strong evolutionary constraint, mounting evidence suggests that viral proteases such as picornavirus 3C, flavivirus NS3, and coronavirus 3CL, are engaged in molecular ‘arms races’ with their targeted host factors, resulting in host- and virus-specific determinants of protease cleavage. In cases where protease-mediated cleavage results in host immune inactivation, recurrent host gene evolution can result in avoidance of cleavage by viral proteases. In other cases, such as recently described examples in NLRP1 and CARD8, hosts have evolved ‘tripwire’ sequences that mimic protease cleavage sites and activate an immune response upon cleavage. In both cases, host evolution may be responsible for driving viral protease evolution, helping explain why viral proteases and polyprotein sites are divergent among related viruses despite such strong evolutionary constraint. Importantly, these evolutionary conflicts result in diverse protease-host interactions even within closely related host and viral species, thereby contributing to host range, zoonotic potential, and pathogenicity of viral infection. Such examples highlight the importance of examining viral protease-host interactions through an evolutionary lens.

## Introduction

**Figure 1:**
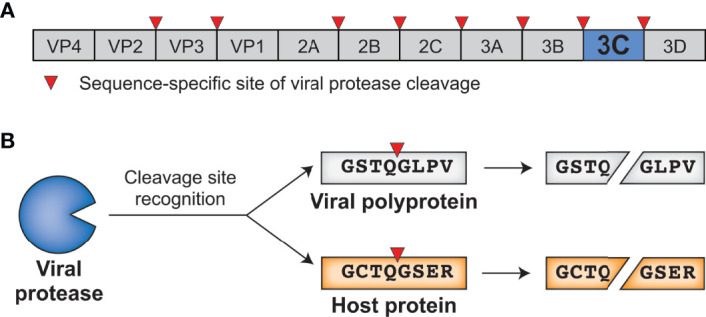
Viral proteases cleave specific sites within the viral polyprotein and host proteins. **(A)** Schematic of an enterovirus (family: *Picornaviridae*) polyprotein, with the position of the 3C protease and sites of 3C-mediated cleavage shown. **(B)** 3C protease recognizes and cleaves viral polyprotein sites and host proteins with the same sequence specificity.

Positive-sense single-stranded RNA [(+)ssRNA, see **Table 1** for glossary of abbreviations] viruses represent the largest group of RNA viruses, spanning 30 divergent viral families that include important human pathogens in *Flaviviridae*, *Picornaviridae*, and *Coronaviridae* such as dengue virus, poliovirus, and SARS-CoV-2 (1). Despite their diversity, many viruses in this group share a common replication strategy: their (+)ssRNA viral genomes are delivered to host cells as a translation-ready mRNA that encodes a multidomain viral polyprotein. Following translation of the viral polyprotein by host ribosomes, one or more embedded viral proteases cleave the polyprotein into individual, functional proteins at numerous sequence-specific positions (**Figure 1A**). Polyprotein cleavage at these specific sites is necessary for sustained virus replication and propagation, making viral proteases an attractive target for development of antiviral therapeutics (2, 3).

**Table 1:** List of abbreviations and alternative names used throughout this review.

Acronym/Abbreviation	Alternative Names	Definition
**General terms**		
ssRNA		Single-stranded RNA
dsRNA		Double-stranded RNA
PRR		Pathogen recognition receptor
ETI		Effector-triggered immunity
LF		Lethal Factor
**Host factors**		
NLRP1	NALP1	NACHT, LRR, and PYD domains-containing protein 1
CARD8	CARDINAL	Caspase Recruitment Domain Family Member 8
eIF4F		Eukaryotic translation initiation factor 4F, composed of subunits eIF4A, EIF4E, and eIF4G
PABP	PABPC1	PolyA binding protein
eIF4A		Eukaryotic translation initiation factor 4A
eIF4G		Eukaryotic translation initiation factor 4G
G3BP1		Ras GTPase-activating protein-binding protein 1
RIG-I	DDX58	Retinoic acid-inducible gene-I-like receptor; DEXD/H-box helicase 58
MDA5	IFIH1	Melanoma differentiation-associated protein 5; interferon-induced with helicase C domain 1
cGAS	MB21D1; C6orf150	Cyclic GMP–AMP synthase; Mab-21 domain containing 1;
IFN		Interferon
ISG		Interferon-stimulated gene
STING	TMEM173	Stimulator of interferon genes; transmembrane protein 173
MAVS	IPS-1, CARDIF, VISA	Mitochondrial antiviral-signaling protein; IFN-β promoter stimulator 1
NF-κB		Nuclear transcription factor kB, often composed of p65 (RelA) and p50 (NFKB1) subunits
NEMO	IKBKG, IKK-gamma	Nuclear transcription factor κB essential modulator
STAT2		Signal transducer and activator of transcription 2
IL-1		Interleukin-1
IκBα	NFKBIA	NF-κB inhibitor alpha
IKK		IκB kinase complex, includes NEMO
** *Picornaviridae* **		
PV		Poliovirus
CVB3		Coxsackievirus B3
FMDV		Foot-and-mouth disease virus
HepA		Hepatitis A virus
EMCV		Encephalomyocarditis virus
** *Coronaviridae* **		
3CL	NSP5; Mpro	3C-like; nonstructural protein 5; Main protease
CoV		Coronavirus
PLP		Papain-like protease
SARS-CoV-2		Severe acute respiratory syndrome-associated coronavirus-2, causative agent on COVID-19
MHV		Murine hepatitis virus
hCoV 229E		Human coronavirus 229E
bCoV HKU4		Bat coronavirus HKU4
hCoV-OC43		Human coronavirus OC43
hCoV-HKU1		Human coronavirus HKU1
PDCoV		Porcine deltacoronavirus
** *Flaviviridae* **		
NS3		Nonstructural protein 3
HCV		Hepatitis C Virus
DENV		Dengue virus
YFV		Yellow fever virus
WNV		West Nile virus
JEV		Japanese encephalitis virus
ZIKV		Zika virus

In addition to their essential role in the viral life cycle, (+)ssRNA viral proteases also cleave host proteins to manipulate host processes, including the host innate antiviral immune response (4). Importantly, host targets are cleaved with the same sequence specificity as sites within the viral polyprotein (**Figure 1B**). These dual roles place viral proteases at the intersection of two opposing selective pressures. On one side, the virus and its polyprotein site targets are under strong pressure to be conserved, as any changes to the protease sequence specificity or protease sites without concomitant changes to the other would be deleterious for viral fitness. On the other side, viral fitness may be expected to benefit from a protease’s ability to adapt to and cleave new host targets, newly evolved sequences in the same host, or divergent sequences in a different host to facilitate cross-species transmission. This type of direct engagement between viral proteases and host factors thus generates an evolutionary conflict where both sides may be driven to adapt in a type of escalating molecular ‘arms race’ (5–8).

**Figure 2:**
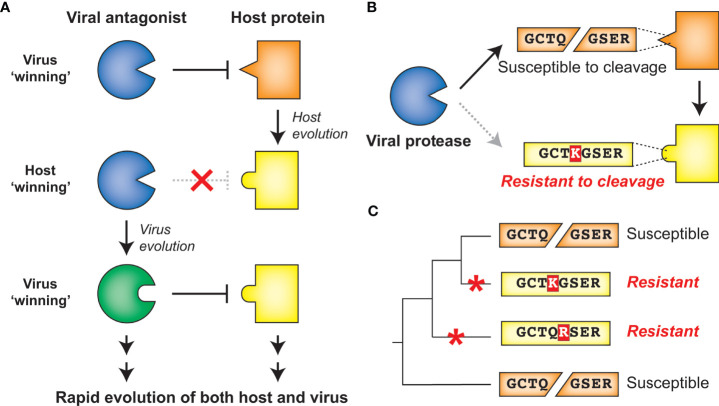
Host-virus evolutionary arms races can be driven by protease-target interactions. **(A)** Host-virus arms races occur when there is direct interaction between host and viral factors, which places evolutionary pressure to select for variants. In this scenario, a viral antagonist recognizes and inactivates a host protein, driving host evolution away from this interaction. The necessity of host target cleavage for virus replication in turn drives evolution of the viral antagonist to reestablish host target recognition. **(B)** Single amino acid changes in the sequence-specific cleavage motif can eliminate cleavage by a viral protease. **(C)** Across a phylogenetic tree, changes can occur recurrently resulting in differential susceptibility between even closely related species. Red asterisks mark the branch in which an amino acid change occurred that alters cleavage susceptibility.

Molecular arms races exist as a result of the competing evolutionary interests of viruses and their hosts. Such competing interests establish an evolutionary equilibrium that is characterized by cyclical adaptations that exemplify a so-called ‘Red Queen’ genetic conflict (9). In these cases, viral adaptations that allow for successful infection of a host will provide a temporary advantage to the virus. However, host adaptations may restore the advantage to the host, applying selection pressure back to the virus (**Figure 2A**). Thus, molecular interactions between viruses and their hosts, particularly those interactions that contribute to potentiation or inhibition of virus replication, are shaped by immense evolutionary pressure on both parties: hosts are driven to both maintain interactions that activate or carry out antiviral defenses and evade virus interactions that prevent these responses, and viruses are driven to do the opposite. The result is recurrent adaptation of both virus and host to promote either virus replication or host antiviral mechanisms, respectively (**Figure 2A**) (5–8). Due to the fact that the direct molecular host-virus interfaces are those that are being remodeled during such molecular arms races, single amino acid changes can change the outcome of these conflicts (5, 7). Indeed, traces of these host-virus conflicts can be detected in host genomes by identifying gene codons that show evolutionary signatures of recurrent diversifying (positive) selection (5, 7, 10). Similarly, viruses are known to adapt during or following cross-species transmission to a novel host, and such adaptations can also be characterized by signatures of positive selection in viral genomes (11–14). Importantly, whether the host has evolved to the virus or the virus has evolved to the host, the resulting genetic and molecular changes determine the host range and pathogenesis of viruses, including influencing the ability of viruses to zoonotically transmit into the human population (7, 8, 13).

Due to the importance of sequence specificity to protease-host interactions, evolutionary arms races at the interfaces of proteases and their targets would be expected to exist. For instance, a single amino acid change in a targeted host protein at a position that is important for sequence-specific protease cleavage could completely reverse cleavage susceptibility (**Figure 2B**). As a result, single lineage-specific changes at any number of positions in the cleavage motif would be expected to alter cleavage susceptibility even among closely related hosts, establishing species-specific host-virus interactions that could drive viral host range (**Figure 2C**). Indeed, while a great deal of research on viral proteases has focused on conserved elements of protease function, emerging evidence suggests that both hosts and viruses are evolving in ways that can impact the host- and virus-specificity of cleavage. Here, we review the host-viral molecular conflicts engaged by the main proteases of flaviviruses, picornaviruses, and coronaviruses to emphasize how proteases of (+)ssRNA viruses act as evolutionary drivers of host innate immunity, and how viral proteases are being shaped by these same molecular conflicts. This evolutionary perspective highlights the importance of viral proteases and their host targets as being an important determinant of viral host range, tissue tropism and pathogenesis, and zoonotic potential of (+)ssRNA viruses.

## Despite Evolutionary Constraints, Main Proteases of (+)ssRNA Viruses Continue to Evolve

**Figure 3:**
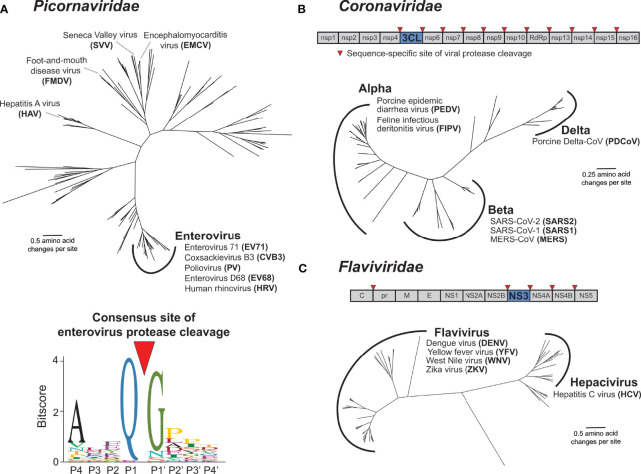
Main proteases in *Picornaviridae, Coronaviridae*, and *Flaviviridae*. **(A)** Phylogenetic tree of available RefSeq *Picornaviridae* 3C protease protein sequences (151 total, top). Names of viruses with human relevance or referenced throughout the text are listed next to their respective genus or singular node. The consensus enterovirus 3C cleavage motif (bottom) as was generated previously (15). The cleavage site is shown flanked by four amino acids upstream (labeled P4 through P1) and four amino acids downstream (labeled P1’ through P4’). **(B)** Schematic of the SARS-CoV-2 (family: *Coronaviridae*) nonstructural (ORF1ab) polyprotein, with the position of the 3CL protease and sites of 3CL-mediated cleavage shown. Phylogenetic tree of available RefSeq *Coronaviridae* 3CL protease protein sequences (64 total). Names of viruses with human relevance or referenced throughout the text are listed next to their respective genus. **(C)** Schematic of the dengue virus (DENV) (family: *Flaviviridae*) polyprotein, with the position of the NS3 protease and sites of NS3-mediated cleavage shown. Phylogenetic tree of available RefSeq *Flaviviridae* NS3 protease protein sequences (68 total). Names of viruses with human relevance or referenced throughout the text are listed next to their respective genus.

Virus-encoded proteases are essential to the life cycle of numerous (+)ssRNA viruses. Newly synthesized viral polyproteins mature into individual, functional proteins *via* a series of cleavage events carried out by virus-encoded and host proteases. For *Picornaviridae* and *Coronaviridae*, the viral cysteine proteases 3C and 3C-Like (3CL) respectively, are responsible for the majority of polyprotein processing events (**Figures 3A, B**) (4, 16–19). Most picornaviruses have six or more 3C cleavage sites throughout the polyprotein (**Figure 1A**), and there is a preference to cleave between a glutamine (Q) in the P1 position and a small residue [e.g. glycine (G) or serine (S)] in the P1’ position (**Figure 3A**) (15, 16, 20). Likewise, coronaviruses (CoVs) have ten or more cleavage sites for the 3CL protease (also known as MPro or nsp5 in several CoVs including SARS-CoV-2) (**Figure 3B**) (17, 18, 21). Numerous other viral families, including members of *Caliciviridae* (e.g. norovirus) (22) and *Potyviridae* (*e.g.* tobacco etch virus) (23) encode a cysteine protease with a similar specificity for cleavage between a Q and a small residue, whereas members of *Togaviridae* (*e.g.* Chikungunya virus) use a cysteine protease with different cleavage specificity (24). Other viral families use a serine protease, including *Flaviviridae*, where the serine protease NS3 processes at least four polyprotein cleavage sites (**Figure 3C**) (25, 26). Here and in subsequent sections, we will predominantly discuss activities of the 3C, 3CL, and NS3 proteases of *Picornaviridae*, *Coronaviridae*, and *Flaviviridae*, respectively, due to their known roles in cleaving mammalian host factors. It is important to point out that because the polyprotein is sequentially processed, and not always to completion, protease activity may also be carried out when 3C, 3CL, or NS3 remains fused or associated with additional viral proteins (27, 28). This is especially true in the *Flaviviridae*, where the NS3 protease usually functions in association with NS2B (in the case of flaviviruses such as dengue and Zika viruses) or NS4A [in the case of hepatitis C virus (HCV)] (26, 29). However, for the sake of clarity, we will subsequently only refer to the protease domains of 3C, 3CL, or NS3. Moreover, many (+)ssRNA viruses encode additional proteases involved in both polyprotein processing and host antagonism, including the 2A protease in some picornaviruses and the papain-like protease (PLP) in coronaviruses (18, 30). Finally, viruses other than (+)ssRNA viruses can encode proteases that are important for polyprotein processing, most notably, the retrovirally-encoded aspartyl protease (31). While all of these additional proteases from (+)ssRNA viruses and retroviruses play important host antagonism roles, and likely shape host and viral evolution, they will not be extensively explored here.

The functions of the (+)ssRNA viral proteases described above are, by definition of being required for completion of the viral life cycle, well conserved. In addition to homology between the proteases themselves, the positions and sequences of the polyprotein cleavage motifs are often similar between members of the same viral family. Indeed, this conservation of polyprotein cleavage motifs has made it possible to compile sequences surrounding the cleavage site from genome sequences alone to generate a consensus motif for the viral protease that can be used to predict host and viral targets (15, 20, 32, 33) (**Figure 3A**). These consensus motifs are often generated using many diverse viruses, relying on the assumption that protease sequence specificity is well conserved among virus species. Interestingly, despite the evolutionary constraint to maintain cleavage across multiple sites in the polyprotein, virus-encoded proteases are substantially divergent across viruses (**Figure 3**). For instance, picornavirus 3C proteases can share less than 20% amino acid sequence identity, despite sharing an overall similar fold and many homologous cleavage sites (15). Similar evolutionary distances are observed with other families of proteases, including *Coronaviridae* 3CL and *Flaviviridae* NS3 (**Figure 3**).

Even with the divergence of protease sequences, protease sequence specificity is expected to be well conserved within closely related viruses given the essentiality of cleaving multiple site-specific polyprotein sites. Surprisingly, there is mounting evidence that this is not the case. For instance, among closely related serotypes of dengue virus (DENV), biochemical substrate profiling has revealed a subtle but clear shift in the NS3 protease cleavage sequence specificity profile (34). This type of in-depth comparative biochemical analysis of other (+)ssRNA proteases has not been conducted, but assays on model substrates have revealed differences in cleavage specificity even among 3C proteases within the Enterovirus genus of *Picornaviridae* (35). Some of the best evidence that protease sequence specificity is changing between related viruses has come from studies using chimeric viruses in which the protease of one virus species is inserted into the backbone of another virus. If such protease swaps result in insufficient or improper cleavage of the polyprotein and reduced viral replication, it would suggest divergence in protease sequence specificity between the parental viruses. For example, among enteroviruses (**Figure 3A**), replacing the poliovirus (PV) 3C protease with 3C proteases from human rhinovirus 14 or coxsackievirus B3 (CVB3) resulted in reduced, changed, or loss of cleavage products (36). Likewise, within the Flavivirus genus of *Flaviviridae* (**Figure 3C**), swapping the protease domain of DENV NS3 for the protease domain of yellow fever virus (YFV) ablates processing of polyproteins containing DENV cleavage sites (37). Additionally, West Nile virus (WNV) NS3 can cleave a polyprotein site in only one of two closely related DENV2 strains, where the only difference is in the residue in the P1’ position (38). While some of these differences may be attributed to the requirement for NS3 proteases to bind to lineage-specific activating cofactors to further augment cleavage specificity (39–47), it is also likely that these changes in cleavage specificity are dependent on non-conserved residues in the binding pocket of the NS3 protease (48). Similarly, within *Coronaviridae*, replication competent chimeric murine hepatitis virus (MHV) could not be recovered when the 3CL protease was replaced with one of many related alpha- or beta-coronavirus proteases including SARS-CoV, hCoV-229E and bat CoV-HKU4 (**Figure 3B**). Only when the MHV 3CL was replaced with the two most closely related beta-coronaviruses, hCoV-OC43 and hCoV-HKU1, could virus be recovered, but with a substantial fitness cost (49). Altogether, these biochemical and chimeric virus studies illustrate that (+)ssRNA viruses have undergone lineage-specific evolution in both their protease sequence specificity as well as their many polyprotein cleavage sites.

## (+)ssRNA Viral Proteases Have Evolved in Conflict With Their Hosts

The above-described changes in protease sequence specificity do not require invocation of adaptation. Indeed, evolutionary drift could result in changes to the viral protease and its cleavage sites, including those that result in loss of fitness for chimeric viruses. However, there is another selective pressure that likely shapes viral protease evolution: the advantage that viruses gain by cleaving host targets. Proteins in multiple cellular processes have been identified as targets of viral proteases, many of which are involved in the host antiviral immune response (4). Many of these host targets are divergent between species, potentially establishing molecular barriers to cross-species transmission. Although the ability to cleave the viral polyprotein is an invariant function of viral proteases, we posit that cleavage of specific host proteins may be selected for during viral evolution, especially during or following cross-species transmission. Indeed, pathogenicity of a mouse-adapted SARS coronavirus required two mutations in 3CL to facilitate rapid, robust virus replication (50). Although it has not been established whether these 3CL changes result in changes in host target cleavage, these data indicate that protease evolution may be required for successful adaptation to a novel host species.

Several excellent reviews have been written describing the diverse host targets that are cleaved by viral proteases (4, 16, 17, 19, 51). In many cases, the described host-virus interaction has focused on a single or a small number of related viral proteases and only a single host species, often humans. Thus, the importance of host and virus diversity in these interactions is often poorly understood. However, evidence is accumulating that viral proteases and their host targets are engaged in species-specific interactions. Below, we highlight such cases in which host and viral diversity alter the outcome of the interaction between host pathways and proteases of picornaviruses, flaviviruses and coronaviruses, illustrating this ongoing molecular arms race.

### Viral Proteases Target Essential Host Processes in a Virus-Specific Manner

Some of the best studied targets of viral proteases, especially from picornaviruses, are involved in well conserved processes such as translation initiation or translation control (52, 53). In many cases, the functional outcome is similar: viral proteases antagonize a host molecular function in a way that benefits the virus. However, the specific host protein or specific site within that host protein can be divergent between different viruses, highlighting differences in protease cleavage specificity between related viruses, as well as the convergence of viral protease cleavage on the same host pathways. Thus, even for host functions that are ‘well conserved’ targets of protease cleavage, there is surprising mechanistic diversity. Below we highlight two such examples in well described targets of picornavirus proteases, but likely many other similar examples exist.

**Figure 4:**
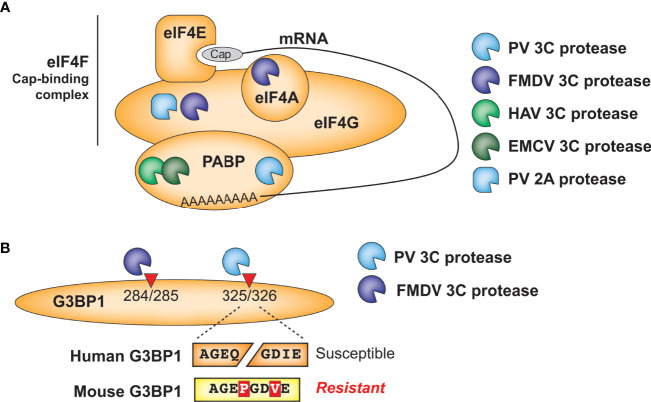
Antagonism of host cellular processes by viral proteases. **(A)** Diverse viral proteases inhibit translation of host mRNA through cleavage of initiation factors and/or poly(A)-binding protein. **(B)** Host and virus species-specific cleavage of the stress granule protein G3BP1 by picornavirus proteases.

Translation of picornavirus mRNAs occurs *via* an internal ribosome entry site (IRES) (54). This bypasses the need to engage with host cap-dependent translation machinery and offers the opportunity to induce a ‘host-shutoff’ of translation of host antiviral proteins while maintaining production of viral proteins. Many picornaviruses inhibit host translation in a protease-dependent manner *via* cleavage of subunits of the eIF4F cap-binding complex, which binds to host mRNA cap structures to establish the initiation complex, or poly-A binding protein (PABP), which binds the 3’ polyA tail of mRNAs and eIF4G to circularize mRNAs for optimal translation initiation (55) (**Figure 4A**). For instance, Foot-and-mouth disease virus (FMDV) 3C cleaves the eIF4G and eIF4A subunits of eIF4F (56). Interestingly, neither hepatitis A (HepA) virus nor encephalomyocarditis virus (EMCV) 3C target eIF4G for cleavage, but both target PABP (57, 58). Convergently, PV also targets PABP, but at a site that is ~100 residues away from the cleavage site of EMCV (59) and additionally uses its 2A protease to cleave eIF4G (60). Despite cleaving different host targets and/or host sites, these interactions all result in host translation shut-off. These data highlight functional conservation, rather than molecular conservation, of picornavirus 3C-mediated inhibition of host translation and suggests that even among related viruses, there are important differences in the viral specificity of host target cleavage.

A similar phenomenon is observed in another well-established target of picornavirus 3C proteases, the stress granule protein G3BP1 (**Figure 4B**). Numerous viruses manipulate stress granule formation for their benefit, as this is a major intersection point between translation control and cellular stress responses (53, 61). Among the mapped cleavage sites in G3BP1, PV 3C cleaves at Q326 (62) while FMDV 3C cleaves at E284 (63), but both of these cleavage events benefit the virus by manipulating stress granule formation. These findings further demonstrate the convergence of 3C cleavage onto the same host target, while highlighting how subtle differences in cleavage specificity can impact viral targeting of host factors. Of note, the P1 and P3’ positions of the PV cleavage site are altered in a way that would prevent cleavage of mouse G3BP1, which is otherwise >90% identical to the human protein (**Figure 4B**). Whether host G3BP1 is cleaved by enteroviruses that infect rodents, and at what site, has yet to be determined.

### Proteins in the Innate Antiviral Immune Response Are Common Targets of Viral Proteases

**Figure 5:**
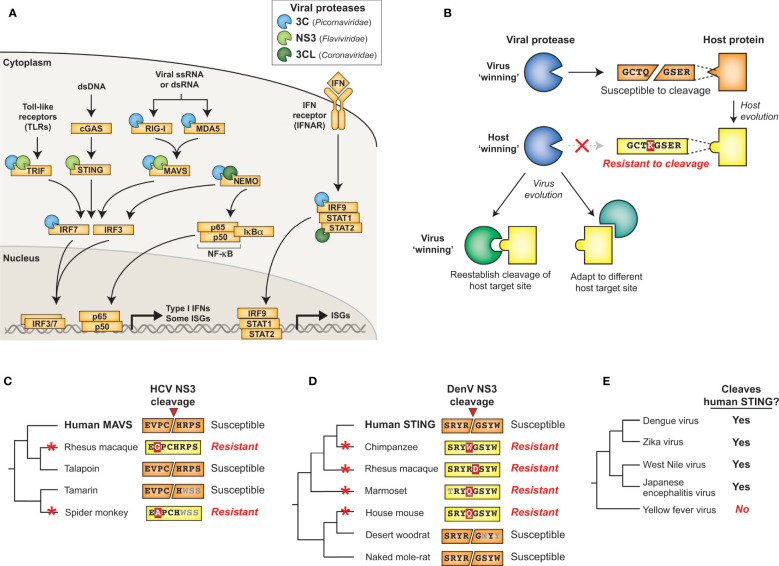
Protease antagonism of IFN induction and signaling pathways. **(A)** Examples of viral proteases that antagonize the innate antiviral immune response, including antagonism of IFN-induction (left) or signaling downstream of IFN (right). **(B)** Model for how protease sequence specificity may be driven to evolve by conflicts with host factors. Following host evolution, or cross-species transmission, viral proteases may no longer be able to antagonize a given host factor. To re-establish host antagonism, the protease can evolve to cut a different sequence at same host site (left) or may evolve to cut a new site elsewhere in the host protein (right). **(C, D)** Evolution of MAVS (64) **(C)** and STING (65) **(D)** across primates and other mammals confers resistance or susceptibility to flaviviral protease cleavage. Red asterisks mark the inferred branch in which an amino acid change occurred that alters cleavage susceptibility. **(E)** Human STING cleavage by flavivirus NS3 proteases is virus species-specific. Data adapted from (66).

n addition to essential cellular processes, proteins in the innate antiviral immune response are common targets of diverse viral proteases. The host antiviral response is initiated when cells detect viral products (**Figure 5A**). Following entry into a host cell, viral nucleic acids can be detected by host pattern recognition receptors (PRRs) such as RIG-I, MDA5, and cGAS. While RIG-I and MDA5 directly detect viral ssRNA or dsRNA as a product of (+)ssRNA virus replication (67–71), the cytosolic DNA sensor cGAS can be indirectly activated *via* virus-induced mitochondrial damage and subsequent release of mitochondrial DNA that can occur during (+)ssRNA viral infection (72). After ligand binding, PRRs recruit a series of adaptor proteins, ultimately resulting in the production and secretion of type I and III interferons (IFN-I and IFN-III) (73). IFN-I and IFN-III are antiviral cytokines that signal in an autocrine or paracrine manner to induce expression of interferon-stimulated genes (ISGs), which act to directly and indirectly inhibit virus replication and establish an antiviral state in the host (74) (**Figure 5A**).

Induction of IFN and subsequent upregulation of ISGs is critical to the host antiviral defense. Therefore, proteins involved in these pathways are common targets of viral antagonism (73), including several that are cleaved by (+)ssRNA viral proteases (**Table 2** and **Figure 5A**). For instance, NS3 from DENV and other flaviviruses can cleave and inactivate STING to prevent sensing of cytoplasmic mitochondrial DNA (65, 75), whereas PV and possibly other 3C proteases cleave RIG-I during infection (76, 96) (**Figure 5A**). Tellingly, many proteases convergently cleave the same host targets. For instance, CVB3 3C and HCV NS3 are both able to cleave MAVS (77, 78), a critical innate immune adaptor for both MDA5 and RIG-I (**Figure 5A**). 3CL proteases from Porcine Epidemic Diarrhea Virus (PEDV), porcine deltacoronavirus (PDCoV), and feline infectious peritonitis virus (FIPV), as well as 3C proteases from FMDV and HepA can also inhibit RIG-I/MDA5 pathways by cleaving nuclear transcription factor κB (NF-κB) essential modulator (NEMO), a bridging adaptor protein involved in activating both NF-κB and interferon-regulatory factor signaling pathways (89–93, 97) (**Figure 5A**). Finally, STAT2, one of the critical transcription factors that transmits the signaling of IFN to ISG production (**Figure 5A**), is cleaved by the 3CL from PDCoV (85), although whether other 3CLs cleave this protein is unknown. Altogether these data show that viral protease-mediated cleavage of innate immune signaling proteins is a common strategy across (+)ssRNA viruses to prevent the antiviral response and promote virus replication.

**Table 2:** Select list of IFN pathway-related targets of (+)ssRNA virus proteases.

Host target	Viral protease	References
STING	NS3 (NS2B3) (ZIKV, JEV, WNV, YFV, DENV)	(65, 66, 75)
RIG-I	3C (PV)	(76)
MAVS	NS3 (NS3-4A) (HCV, GBV-B)	(64, 77–82)
	3C (CVB3, SVV)	
Riplet	NS3 (NS3-4A) (HCV)	(83)
MDA5	3C (FMDV)	(84)
STAT2	3CL (PDCoV)	(85)
TRIF	NS3 (NS3-4A) (HCV)	(78, 81, 86–88)
	3C (CVB3, SVV, EV68)	
	3CD (HAV)	
NEMO	3CL (PEDV, FIPV, PDCoV)	(89–93)
	3C (FMDV, HAV)	
IRF7	3C (EV68)	(94)
IRF9	3C (EV71)	(95)

Viral abbreviations are as follows: ZIKV, Zika virus; JEV, Japanese encephalitis virus; WNV, West Nile virus; YFV, Yellow fever virus; DENV, Dengue virus; PV, Poliovirus; HCV, Hepatitis C virus; GBV-B, GB virus B or Pegivirus B; CVB3, Coxsackievirus B3; SVV, Seneca Valley virus; FMDV, Foot and mouth disease virus; PDCoV, Porcine deltacoronavirus; EV68, Enterovirus D68; HAV, Hepatitis A virus; PEDV, Porcine epidemic diarrhea virus; FIPV, Feline infectious peritonitis virus.

Many proteins in the innate antiviral immune response are rapidly evolving within and between host populations (98–100). One potential consequence of these host changes is that a cleavage site for a viral protease may be present in one host but not another. If there is strong selection for the virus to restore antagonism of that host function, there would be selection for viral proteases that would change the sequence specificity of host target cleavage to either restore cleavage of the original site, cleave another site on the host protein, or cleave another protein in the host pathway (**Figure 5B**). Such an evolutionary model can be used to understand the genetic bases for host- and viral-specificity of protease cleavage. For many of the known interactions between host immunity proteins and viral proteases, there is little information on how host and viral evolution shapes the outcome. However, analyses on two host targets, described in more detail below, provide evidence for an arms races between host immunity proteins and viral proteases.

### MAVS and STING Have Evolved in Conflict With Viral Proteases

One well-characterized instance of viral proteases shaping host gene evolution is in HCV NS3 protease antagonism of the host protein MAVS. MAVS serves as a critical signaling node to integrate signals from the nucleic acid sensors RIG-I and MDA5 to downstream IFN production (**Figure 5A**). Early observations indicated that MAVS cleavage by HCV NS3 was site specific and important for viral evasion of the immune system (101). Subsequent evolutionary analyses revealed that one residue within the HCV cleavage site in MAVS has evolved under recurrent positive selection, suggestive that MAVS evolution has been shaped by NS3 antagonism (64). Variation at this site across primates affects susceptibility to cleavage by HCV NS3. Importantly, primate MAVS proteins that have evolved resistance to cleavage retain a functional IFN response during HCV infection, providing a potential explanation for the restricted host range of HCV (**Figure 5C**) (64). This work also identified a site evolving under positive selection that is known to be antagonized by the CVB3 3C protease, and variation at this site across primates could also alter protease-mediated antagonism and antiviral signaling through MAVS (64, 78).

Another adaptor protein that connects nucleic acid sensing to the IFN response is STING, which operates downstream of the cytoplasmic DNA sensor cGAS (**Figure 5A**). Originally described as a species-specific target of DENV NS3 cleavage (65, 75), STING has evolved under positive selection in primates and the NS3 cleavage site within STING contains several amino acid differences across primates that alter the outcome of cleavage (**Figure 5D**) (65). Expanding this analysis to a broader panel of mammals, the NS3 site of cleavage in human STING has evolved to be cleavage resistant in mice, pigs, and ground squirrels, whereas naked mole rat and desert woodrat are susceptible to cleavage (65) (**Figure 5D**). Interestingly, differences in protein sequences that affect cleavage do not just occur between host species; polymorphisms within a host can also alter the ability of a viral protease to cleave a given target. Evidence of this process can be observed in human STING polymorphisms, where the three most common human STING haplotypes are differentially cleaved by DENV NS3 (102). Not only is host diversity important, but viral diversity is as well. For instance, ZIKV, DENV, JEV, and WNV NS2B3 can cleave human but not mouse STING, whereas YFV NS3 cannot cleave STING from either species (66) (**Figure 5E**). Additional work to identify more divergent flaviviral protease interactions will further define evolution of STING antagonism.

## Immune Sensors of Viral Protease Activity: Who Is Chasing Whom?

Cleavage of host proteins by viral proteases often inactivates the host protein and results in a fitness advantage for the virus. In these cases, host evolutionary signatures reveal adaptations that are presumed to evade cleavage. However, another possibility exists, in which the host protein can sense the presence of the viral protease in the cytoplasm through an evolved sequence that mimics the viral polyprotein cleavage site. Sensing of pathogen-encoded activities such as toxins and effector enzymes, known as effector-triggered immunity (ETI), is well-described in plants but is also emerging as an important immune mechanism in animals (103–106). Three such signaling pathways, described below, are known to detect the main protease activity of human viruses.

### NLRP1 Mimics Diverse Picornaviral 3C Cleavage to Trigger Inflammation

**Figure 6:**
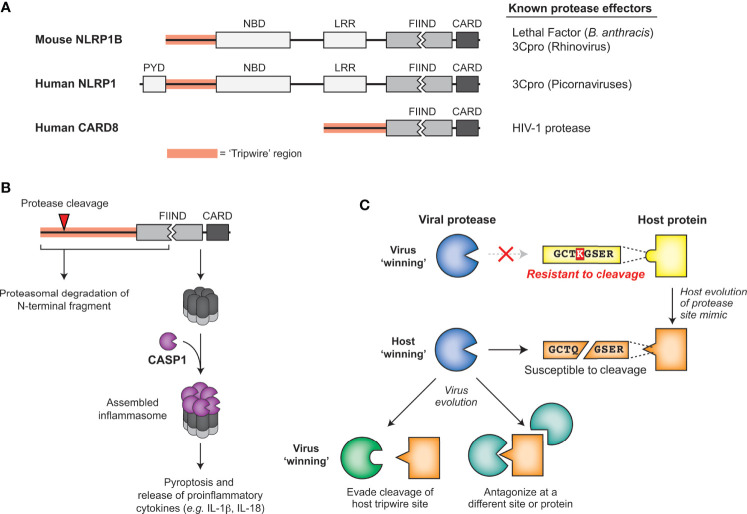
Sensing of pathogen-encoded protease activities by host ‘tripwires’. **(A)** NLRP1 and CARD8 serve as effector-triggered immunity (ETI) sensors to detect cleavage by viral proteases. Schematic of mouse NLRP1B, human NLRP1, and human CARD8, highlighting the tripwire region (left) and the known protease effectors (right). **(B)** Model for how protease cleavage initiates functional degradation of the N-terminal region of inflammasome activators. Activation recruits and activates caspase-1, which cleaves multiple host proteins, including processing proinflammatory cytokines such as IL-1β, into their mature, bioactive form. **(C)** Model for how evolution of host protease site mimics may drive viral protease evolution to either evade cleavage of the host tripwire or antagonize the host in other parts of the protein or pathway.

One of the best described cases of mammalian ETI involves NLRP1 (NACHT, LRR, and PYD domains-containing protein 1; **Figure 6A**), a critical sensor for the innate immune complex known as the inflammasome. Mouse NLRP1B was identified in a genetic screen as a determinant of differential susceptibility between mouse strains to Lethal Toxin, a virulence factor responsible for the major pathologies seen during infection by the bacterial pathogen *Bacillus anthracis* (107). Further research identified that NLRP1B was a target of cleavage by the secreted bacterial protease component of Lethal Toxin, termed Lethal Factor (LF). Interestingly, mice with a cleavage-susceptible variant of NLRP1B were protected from *B. anthracis* challenge, indicating that cleavage of NLRP1B was immunologically protective (108, 109). The mechanism by which this occurs, termed ‘functional degradation’ (110, 111), depends on the FIIND domain encoded within NLRP1B, which undergoes a constitutive self-cleavage event (known as ‘auto-processing’) such that the N-terminal domains and C-terminal CARD-containing fragment of NRLP1B exist as two distinct, noncovalently associated polypeptides (112, 113) (**Figure 6B**). Once LF cleaves upstream of the FIIND domain in NLRP1B, the released product has a new N-terminus that is recognized by the N-end rule cellular machinery and targets it for proteasome-mediated degradation. However, as a result of the break in the polyprotein backbone within the FIIND domain, proteasome-mediated degradation of NLRP1B ceases after degrading the N-terminal domains, leaving the bioactive C-terminal fragment intact and able to assemble into an active inflammasome (**Figure 6B**) (110, 111). The unusual domain architecture of NLRP1B thus facilitates the mounting of the inflammasome response upon proteolytic cleavage of the N-terminus.

LF cleaves within the rapidly evolving ‘tripwire’ region of mouse NLRP1B but fails to cleave or activate human NLRP1. Interestingly, human NLRP1 has an analogous rapidly-evolving ‘tripwire’ region (**Figure 6A**), and cleavage of human NLRP1 *via* an engineered tobacco etch virus (TEV) protease cleavage site can activate the inflammasome (114). These data suggested that human NLRP1 may also detect pathogen-encoded proteases and activate the inflammasome *via* a functional degradation mechanism. Indeed, we and others recently identified that human NLRP1 recognizes picornavirus 3C protease activity and serves as a tripwire for inflammatory cell death and downstream inflammatory signaling (15, 115). During enterovirus infection, 3C cleavage of NLRP1 results in assembly of the active inflammasome and subsequent pro-inflammatory cytokine release (15, 115), including in human primary airway epithelial cells (115). Interestingly, based on phylogenetic analyses, the 3C-protease site mimic in this specific region of NLRP1 only evolved in the primate lineage, and is only cleavable in some primates (15). Differences across simian primates and a SNP within the human population prevent cleavage and inflammasome activation (15). Although mice lack this human-aligned cleavage site, we discovered a similar phenomenon where picornavirus 3C proteases cleave NLRP1B at different sites to activate the inflammasome in a virus- and mouse-strain-specific manner (15).

In addition to host diversity, viral diversity also determines NLRP1 cleavage. While all enteroviruses cleave the same site within NLRP1 and activate the inflammasome, other picornaviruses cleave NLRP1 at different sites within the N-terminal domain or do not cleave NLRP1 (15). For instance, the 3C protease of EMCV does not cleave NLRP1, and resultingly no activation of the NLRP1 inflammasome was observed upon EMCV infection (15). As numerous sites in the protease-sensing N-terminal region of NLRP1 are evolving under positive selection (114), other independently evolved tripwire sites within NLRP1 may sense divergent 3C or other viral proteases.

### Intracellular HIV-1 Protease Activity Triggers Inflammation *via* CARD8

Another inflammasome mediator, CARD8, is known to share the unusual C-terminal domain structure critical for the sensing mechanism of NLRP1 – the FIIND domain followed by a CARD domain (**Figure 6A**) (116, 117). In addition to these domain similarities, CARD8 inflammasome assembly can also be activated by the same small molecules as NLRP1 (117). Such similarities initially suggested that CARD8 could also be activated using a functional degradation model to act as a tripwire sensor of pathogen-encoded activities (116). Indeed, the protease of human immunodeficiency virus 1 (HIV-1) can cleave and activate the CARD8 inflammasome in an activation mechanism that resembles NLRP1 (118). While HIV-1 protease is normally important for cleaving viral polyproteins in the maturing capsid, treatment with specific non-nucleoside reverse transcriptase inhibitors (NNRTIs) can result in protease activity in the cytoplasm (119). Under these NNRTI treatment conditions, HIV-1 proteases from four prevalent HIV-1 subtypes cleave CARD8 and activate the inflammasome, resulting in pro-inflammatory cytokine release and influencing clearance of latent HIV-1 in primary CD4+ T cells (118). While the extent to which host evolution or evolution of other viruses influences the activation of the CARD8 system remains unknown, these findings reveal a broader role for host encoded tripwires for viral proteases that can activate a robust immune response using mimicry of viral protease cleavage sites.

### 3C-Mediated Cleavage of a Regulator of NF-kB Triggers Apoptosis

‘Tripwire’ mechanisms such as NLRP1 and CARD8 rely on a specific elegant, but rare, domain architecture that allows for coupling of a cleavage event to generation of a bioactive signaling molecule. An additional mechanism for sensing of viral proteases arises from the intricate ways that the innate immune response is negatively regulated. For instance, downstream of NLRP1 and CARD8, mature inflammatory cytokines are detected by the IL-1 receptor to activate the transcription factor NF-κB, which can amplify the inflammatory response (120). NF-κB is an essential transcription factor involved in many innate immune pathways and can mediate a variety of downstream responses depending on the input stimuli (121), including pro- or anti-apoptotic responses (122). Within the cytoplasm, the NF-κB heterodimer, composed of the Rel family proteins p65 and p50, remains bound and inactive by members of the inhibitor of κB (IκB) family, including IκBα (**Figure 5A**). In response to cytokines such as IL-1β, IκB kinase (IKK) family proteins phosphorylate IκB proteins, releasing the active transcription factor to translocate into the nucleus (120). A previous study demonstrated that IκBα senses CVB3 3C protease activity (123). The 3C protease was shown to cleave IκBα, producing a fragment that stably complexes with p65 and translocates to the nucleus. This stable complex blocks NF-κB transcriptional activation, resulting in increased cell apoptosis and decreased viral replication (123). Thus, cleavage of IκBα may have evolved as another way to sense viral protease activity and induce cell death to prevent further virus propagation. Many viral proteases are known to cleave proteins in the NF-κB pathway (**Table 2**). Additional characterization of these virus-host interactions may reveal additional antiviral mechanisms associated with this critical immune pathway.

### Evolutionary Advantages of ETI

In the continual evolutionary conflict between viruses and their hosts, cleavage mimicry encoded in NLRP1, CARD8 and NF-kB serve as examples of a successful strategy emerging in host organisms to exploit highly constrained pathogenic processes. Viruses are known to use molecular mimicry to antagonize or subvert the host immune response (124). In the cases of ETI described above, the host is turning the tables and using mimicry of viral protease cleavage sites to support the antiviral response. Rather than mimicry of entire proteins or protein domains, mimicry of these cleavage sites as ‘short linear motifs’ (SLIMs) require only a small number of amino acids to hijack the highly conserved protease activity (125, 126). In order to avoid these ‘tripwires’ and negative regulators of the immune response, these viruses must either evolve their respective main proteases along with all affiliate cleavage sites or antagonize the process some other way (**Figure 6C**). Supporting this idea, 3C proteases from some picornaviruses cleave NLRP1 but do not activate the NLRP1 inflammasome, suggesting that 3C proteases have evolved to evade detection by NLRP1 by antagonizing NLRP1 function elsewhere (15). We expect that this work may lead to the discovery that protease-driven ETI strategies may have evolved more broadly at other sites of host-pathogen conflicts.

## Discussion

The proteases of (+)ssRNA viruses have multiple roles in establishing and maintaining virus infection within a host. First and foremost, virally-encoded proteases cleave numerous sequence-specific sites within the viral polyprotein, which is essential for completion of the viral replication cycle. As a consequence of this essential activity, the ability of proteases to evolve novel sequence specificity is highly constrained. However, viral proteases also serve to manipulate numerous host processes in the infected cell through site-specific cleavage of host targets. In this context, changes in protease sequence specificity would allow the virus to cleave new host targets that might benefit the virus, or avoid cleaving host targets that are detrimental to the virus. It is at this intersection that viral proteases are engaged in evolutionary ‘arms races’ with the host, resulting in varied interactions across viral and host species and across evolutionary time. Several examples, including virus-specific cleavage of essential mRNA translation machinery and host-specific evasion of cleavage of innate antiviral immune components, highlight the consequences of these evolutionary conflicts. More recently, the discovery of host-encoded effector-triggered immunity (ETI) sensors such as NLRP1 and CARD8 suggest that host mimicry of viral protease cleavage sites is an efficient strategy to detect the cellular activity of viral proteases.

The extent to which viral protease evolution, and host target diversity, shape viral host range and pathogenesis remains unknown and is an exciting area of future research. The majority of characterized protease-host interactions have been described for a single virus against a single host, leaving open the opportunity for more detailed exploration of the evolutionary dynamics of these interactions. Indeed, examples such as cleavage of host proteins such as MAVS, STING, and NLRP1 highlight the insights that can be gained from additional analyses of host and viral diversity in these interactions. Likewise, future studies aiming to discover additional host targets of viral proteases, especially those that may be cleaved in a virus-specific manner, will advance our knowledge of the ways that protease-host interactions shape viral phenotypes. Finally, ETI sensors such as NLRP1 and CARD8 may represent just the start of host proteins that mimic viral protease cleavage sites to induce an immune response. Further studies aimed to identify ETI mechanisms against both viral and other pathogen-encoded proteases will likely continue to reveal novel mechanisms and evolutionary principles of the host innate immune response.

## Author Contributions

All authors discussed relevant literature. BT, EF, and MD wrote the first draft. All authors contributed to figure generation and editing the manuscript. All authors contributed to the article and approved the submitted version.

## Funding

This work was supported by grants from the National Institutes of Health (R35 GM133633), Pew Biomedical Scholars Program, Hellman Fellows Program, and Burroughs Wellcome Investigators in the Pathogenesis of Infectious Disease Program to MD and an NIH T32 grant (GM007240) to BT.

## Conflict of Interest

The authors declare that the research was conducted in the absence of any commercial or financial relationships that could be construed as a potential conflict of interest.

## Publisher’s Note

All claims expressed in this article are solely those of the authors and do not necessarily represent those of their affiliated organizations, or those of the publisher, the editors and the reviewers. Any product that may be evaluated in this article, or claim that may be made by its manufacturer, is not guaranteed or endorsed by the publisher.
